# The Peptide Fractions of Cheddar Cheese Made with *Lactobacillus helveticus* 1.0612 Play Protective Effects in H_2_O_2_-Induced Oxidative-Damaged Caco-2 Cells Models

**DOI:** 10.3390/foods12142790

**Published:** 2023-07-22

**Authors:** Wanshuang Yang, Xiuxiu Zhang, Meng Sun, Yang Jiao, Xiaodong Li, Lu Liu, Zhong Wang

**Affiliations:** 1 College of Food Science, Northeast Agricultural University, No. 600 Changjiang St., Xiangfang Dist., Harbin 150030, Chinawz1302353461@163.com (Z.W.); 2 Key Laboratory of Dairy Science, Ministry of Education, Northeast Agricultural University, No. 600 Changjiang St., Xiangfang Dist., Harbin 150030, China

**Keywords:** oxidative damage, Caco-2 cells, cheddar cheese, peptide fractions, cytoprotective effect, metabolomics

## Abstract

In this study, water-soluble peptide (WSP) fractions of cheddar cheese made with *Lactobacillus helveticus* 1.0612 were purified into WSP-Ⅰ (<3 kDa), WSP-Ⅱ (3–10 kDa), and WSP-Ⅲ (>10 kDa). The protective effects of WSP, WSP-Ⅰ, WSP-Ⅱ, and WSP-Ⅲ fractions against oxidative stress in Caco-2 cells were assayed, and the cytoprotective mechanism of WSP-Ⅰ on cells oxidative damage was elucidated via metabolomics. The results showed that all four peptide fractions were able to attenuate the decrease in cell viability caused by oxidative stress and also could reduce the production of reactive oxygen species and malondialdehyde caused by oxidative stress, and increased cellular catalase and superoxide dismutase activities, thereby enhancing cellular antioxidant capacity. The WSP-Ⅰ fraction with the highest protective effect was used for metabolomics analysis, and 15 significantly different metabolites were screened. Functional pathway analysis revealed that the protective effect of the WSP-I fraction was related with nine metabolic pathways and weakened the metabolic disorders caused by H_2_O_2_ via regulating energy metabolism and amino acid metabolism. All in all, peptide fractions of cheddar cheese showed a cytoprotective effect through improved cellular metabolism.

## 1. Introduction

Reactive oxygen species (ROS) are essential parts of living organism cells and play a dual role in the physiological processes of cell signal transduction and homeostasis [1,2]. However, excessive accumulation of ROS may cause oxidative stress and consequently lead to the initiation and development of various chronic diseases such as cardiovascular disease, intestinal diseases, diabetes, Alzheimer’s disease, atherosclerosis, arthritis, and inflammation [3,4,5,6]. In addition, ROS are also the main factor leading to food corruption and deterioration, which will lead to bad flavor and carcinogens [7,8]. Therefore, in order to prevent or alleviate food from being damaged by ROS and protect the human body from related oxidative damages, antioxidants have been widely used in the food industry [1]. Food-derived natural antioxidants have drawn more interest from researchers due to their safety and widespread nature, and consumption of food-derived antioxidant compounds has been suggested to enhance the antioxidant system to resist oxidative stress [9,10,11,12,13]. Many protein hydrolysates and peptides of food sources have been investigated, including milk, eggs, grains, and fish, and have been considered beneficial for their protective roles in chronic diseases associated with oxidative stress [14,15,16,17]. 

As a natural food, milk has received a great deal of attention due to its beneficial effects on human health. From this perspective, hydrolysates of milk protein are gradually being used in research to reduce the risk of chronic diseases related to oxidative stress [18]. Cheese is one of the most important dairy products in the world. There are a variety of proteolytic systems in cheese. During the ripening time, the protein in cheese is hydrolyzed to produce a large number of bioactive peptides [7,19,20]. These peptides have been shown to have antioxidant properties [21], such as DPPH and ABTS free radical scavenging activity. In addition, studies have found that milk-derived peptides have a certain protective effect on oxidative-damaged cells [6,16]. Huma et al. [4] evaluated the effect of cheddar cheeses on human colon adenocarcinoma Caco-2 cell lines and found that peptide fractions from cheddar cheeses decreased the content of intracellular ROS. Tonolo et al. [22] showed that milk-derived bioactive peptides exhibited a protective effect against oxidative stress in Caco-2 cells. *Lactobacillus helveticus* is a probiotic with one to four cell-envelope proteases that are specific for casein cleavage. Studies have found that *Lactobacillus helveticus* can improve the proteolysis and antioxidant activity of cheese during the cheese ripening [23]. Yang et al. [24] evaluated the effect of *Lactobacillus helveticus* on the antioxidant activity of cheddar cheese. The results showed that cheese with *Lactobacillus helveticus* added had higher antioxidant activity compared to cheese made without *Lactobacillus helveticus*. However, there have been relatively few studies on the effects of cheese made with *Lactobacillus helveticus* on oxidative damage of cells.

Cellular metabolomics is an important research tool in cellular biochemistry. Cellular metabolomics has been widely used for the determination of small molecular metabolites in cells [25,26] and can provide information about the metabolic pathways of cells [27]. The in vitro antioxidant activity of water-soluble peptide fractions of cheese has been extensively studied [28,29]. However, the cytoprotective effect and metabolomics analysis of water-soluble peptide fractions of cheese in human intestinal cells are seldom reported [4,7,30]. We have determined that cheddar cheese made with *Lactobacillus helveticus* 1.0612 showed in vitro antioxidant activity in previous studies [24]. Therefore, the aim of this study was to evaluate the cytoprotective effect of the water-soluble peptide fractions of cheddar cheese made with *Lactobacillus helveticus* 1.0612 against oxidative stress induced by H_2_O_2_ in Caco-2 cells and further reveal the metabolomics mechanism of the protective effect of it on oxidative-damaged Caco-2 cells. 

## 2. Materials and Methods

### 2.1. Materials

3-(4,5-dimethylthiazol-2-yl)-2,5-diphenyltetrazolium bromide (MTT) was purchased from Biotiopped (Beijing, China). 2′,7′-dichlorofluorescein diacetate (DCFH-DA) and ammonium acetate were purchased from Sigma (Oakville, ON, Canada). The human colon adenocarcinoma Caco-2 cell lines were obtained from the Northeast Agricultural University (Heilongjiang, China). Dulbecco’s modified Eagle medium (DMEM) was purchased from Hyclone (Beijing, China), and fetal bovine serum (FBS) was purchased from Sijiqing (Zhejiang, China). Dimethyl sulfoxide (DMSO), 0.25% trypsin–EDTA, nonessential amino acid (100×) and penicillin–streptomycin (100×) were obtained from Solarbio (Beijing, China). Malondialdehyde (MDA), catalase (CAT), superoxide dismutase (SOD) assay kits, and PMSF RIPA were purchased from the Beyotime Biotech Inc (Shanghai, China). Acetonitrile was purchased from Merck (Darmstadt, Germany).

### 2.2. Experimental Design

Firstly, the water-soluble extract was ultrafiltered by Millipore centrifugal concentrators with molecular-weight cut-off membranes (10 kDa and 3 kDa), and the four peptide fractions after ultrafiltration were collected. Secondly, H_2_O_2_-induced oxidative-damaged Caco-2 cells models was used for cell test. The cell survival rate was determined using the MTT method, and the fluorescence intensity of the cells was observed using the DCFH-DA fluorescence probe. A malondialdehyde kit, superoxide dismutase kit, and catalase kit were used to determine the content of malondialdehyde, superoxide dismutase activity, and catalase activity in the cells. Finally, based on the above experimental results, the peptide fraction with the best protection was selected for metabolomics. OPLS-DA and KEGG pathway enrichment analyses were used to identify significant differential metabolites and related metabolic pathways in the extracellular metabolism of cells during H_2_O_2_ damage and peptide fraction protection processes.

### 2.3. Cheddar Cheese Manufacture

Cheddar cheeses were manufactured according to the method described by Yang et al. [24]. The commercial strains *Lactococcus lactis* subsp. *Cremoris* and *Lactococcus lactis* subsp. *lactis* were used as a starter culture. After pasteurization of raw milk, 1.5% (*v*/*v*) starter cultures and 1.2% (*v*/*v*) *Lactobacillus helveticus* 1.0612 were added to milk for fermentation. Calf rennet was added to the mixture, which had been fermenting for 40 min, to coagulate the milk. After coagulation, the curd was cut into blocks, and the whey was discharged, and then the curd was marinated, pressed, vacuum-sealed, and kept mature at 2–8 °C. According to the previous research results of our research group [24], the cheese of the 5th month was used in this study.

### 2.4. Preparation of Water-Soluble Peptide Fractions of Cheddar Cheese 

Water-soluble peptide (WSP) fractions of cheddar cheeses were prepared according to Yang et al. [24]. A 50 g portion of cheese was grated and homogenized with 200 mL of deionized water at 1000 rpm for 5 min. The homogenized mixture was heated in a water bath at 40 °C for 1 h and then centrifuged at 4000× *g* for 10 min at 4 °C. The supernatant was gathered and filtered through No. 42 Whatman filter paper. The permeate was filtered through a 0.22 syringe filter and then purified by Millipore centrifugal concentrators with molecular-weight cut-off membranes (10 kDa and 3 kDa) (Millipore, Billerica, MA, USA) by centrifugation at 4000× *g* for 20 min at 4 °C. The permeate of a 0.22 syringe filter is initially filtered through a 10 kDa membrane to generate a retentate (MW > 10 kDa), and then the 10 kDa permeate is further filtered through a 3 kDa membrane into two parts of 3 kDa retentate (3–10 kDa) and 3 kDa permeate. Four peptide fractions, WSP-Ⅰ (<3 kDa), WSP-Ⅱ (3–10 kDa), WSP-Ⅲ (>10 kDa), and WSP of cheddar cheese were collected and freeze-dried and stored at −20 °C for further use. 

### 2.5. Cell Culture

Cell culture operation was performed according to the method of Tonolo et al. [31] and Guo et al. [32]. Caco-2 cell lines were cultivated in 25 cm^2^ flasks in DMEM supplemented with 10% FBS, 1% L-glutamine, 1% nonessential amino acid, and 1% penicillin–streptomycin at 37 °C in a humidified atmosphere containing 5% CO_2_ and 95% air. Then, 0.25% trypsin–EDTA was added to digest the cells for passage and subsequent experiments when the cells were at 70–80% of the confluence of the culture flask area. The cells used in this study were between 30 and 60 passages.

### 2.6. Cell Viability

The MTT assay was used to assess the cell viability [6,33]. Caco-2 cells were seeded in 96-well plates (Corning Inc., NYC, USA) at a density of 1 × 10^4^ cells per well and incubated in a humidified atmosphere (95%) and 5% CO_2_ for 24 h. After cell adhesion and growth, different concentrations of WSP-Ⅰ, WSP-Ⅱ, WSP-Ⅲ, and WSP (0.1, 0.2, 0.3, 0.4, 0.5 mg/mL) or H_2_O_2_ (0.2–1.1 mM) were added to the cell culture plate for incubation for 24 h, and then the liquid in the cell culture plate was removed and PBS was added for cell washing. Next, 50 µL of MTT solution (0.1 mg/mL) were added to each cleaned cell well and incubated at 37 °C for 2 h. After incubation, the supernatant was aspirated and discarded, and then 100 µL of DMSO were added to dissolve formazan crystals. The absorbance was measured at 570 nm by a multifunctional microplate reader (INFINITE M200 PRO) after 30 min.

### 2.7. Estimation of Intracellular ROS Levels 

Caco-2 cells in 96-well plates with a density of 1 × 10^4^ cells per well were incubated in complete medium [34]. After cell adhesion and growth, WSP-Ⅰ, WSP-Ⅱ, WSP-Ⅲ, WSP, DMEM, and H_2_O_2_ were added to the 96-well plate according to experimental grouping conditions and then incubated for 24 h. After incubation, the supernatant was discarded and 100 µL of 10 µM DCFH-DA were added to the cell well plate for incubation. After 4 h, the cells were washed three times with PBS and incubated with FBS-free DMEM. The fluorescence intensity was monitored using a multifunctional microplate reader with an excitation wavelength of 485 nm and an emission wavelength of 525 nm.

### 2.8. Assay of Intracellular Content of MDA and Activity of SOD and CAT

Caco-2 cells were seeded in 6-well cell plates at concentrations of 1 × 10^5^ cells per mL. After treatment with WSP-Ⅰ, WSP-Ⅱ, WSP-Ⅲ, WSP (0.4 and 0.5 mg/mL), FBS-free DMEM, and 900 µM of H_2_O_2_, the cells were lysed using 1 mM PMSF RIPA lysis buffer at 4 °C for 15 min and centrifuged at 5000× *g* for 10 min to obtain the supernatant [34]. The supernatant was used for biochemical testing. The levels of superoxide dismutase (SOD), catalase (CAT), and malondialdehyde (MDA) were measured with commercially available kits. All operations were in complete compliance with the manufacturer’s instructions. SOD was measured at 560 nm, CAT at 520 nm, and MDA at 532 nm.

### 2.9. Determination of Metabolomics 

#### 2.9.1. Cell Sample Collection and Extraction

Caco-2 cells treated with the WSP-Ⅰ fraction were used for metabolomics determination. After treatment with WSP-Ⅰ, FBS-free DMEM, and 900 µM of H_2_O_2_, the cell culture fluid was collected and stored at −80 °C for metabolomics determination. After the cell culture fluid sample was slowly thawed at 4 °C, it was added to a precooled methanol/acetonitrile/water solution (2:2:1, *v*/*v*), vortexed, and then underwent an ultrasound for 30 min at low temperature. The ultrasound mixture was left to stand for 10 min at −20 °C and then centrifuged at 14,000× *g* for 20 min at 4 °C to collect the supernatant. The supernatant was dried in vacuum and then redissolved with 100 µL of acetonitrile aqueous solution (acetonitrile: water= 1:1, *v*/*v*) for mass spectrometry.

#### 2.9.2. Metabolomics Analysis

The metabolomics analysis was performed according to the method of Yao et al. [35] and Shao et al. [36] with modification. The Agilent 1290 Infinity LC Ultra-High-Performance Liquid Spectroscopy (UHPLC) HILIC column was used for the separation of the sample. Column temperature: 25 °C; flow rate: 0.5 mL/min; injection volume: 2 µL. The mobile phase consisted of A (water + 25 mM ammonium acetate + 25 mM ammonia) and B (acetonitrile). The gradient elution procedure was as follows: 0–0.5 min, 95% B; 0.5–7 min, B changes from 95% to 65%; 7–8 min, B changes from 65% to 40%; 8–9 min, B was maintained at 40%; 9–9.1 min, B changes from 40% to 95%; 9.1–12 min, B was maintained at 95%. The sample was placed in the 4 °C autosampler during the entire analysis. 

The AB Triple TOF 6600 mass spectrometer was used to collect the primary and secondary spectra of the samples. The ESI source conditions after HILIC chromatographic separation were as follows: Ion Source Gas1 (Gas1): 60; Ion Source Gas2 (Gas2): 60; Curtain gas (CUR): 30; source temperature: 600 °C; Ion Spray Voltage Floating (ISVF) ± 5500 V (both positive and negative modes); TOF MS scan m/z range: 60–1000 Da, product ion scan *m*/*z* range: 25–1000 Da, TOF MS scan accumulation time 0.20 s/spectra, product ion scan accumulation time 0.05 s/spectra. The secondary mass spectrum was acquired by information-dependent acquisition (IDA) and with the high-sensitivity mode. Declustering potential (DP): ±60 V (both positive and negative modes); Collision Energy: 35 ± 15 eV; IDA settings are as follows: Exclude isotopes within 4 Da; Candidate ions to monitor per cycle: 10.

### 2.10. Statistical Analysis

All data are expressed as mean ± standard deviation. Statistical analysis was performed by one-way analysis of variance (ANOVA) followed by LSD test at *p* < 0.05, *p* < 0.01, and *p* < 0.001 using SPSS 26.0. SIMCA and Metaboanalyst 5.0 were used for data analysis in metabolomics.

## 3. Results and Discussion

### 3.1. Influence of Cheese Peptide Fractions on Viability of Caco-2 Cells 

**Figure 1:**
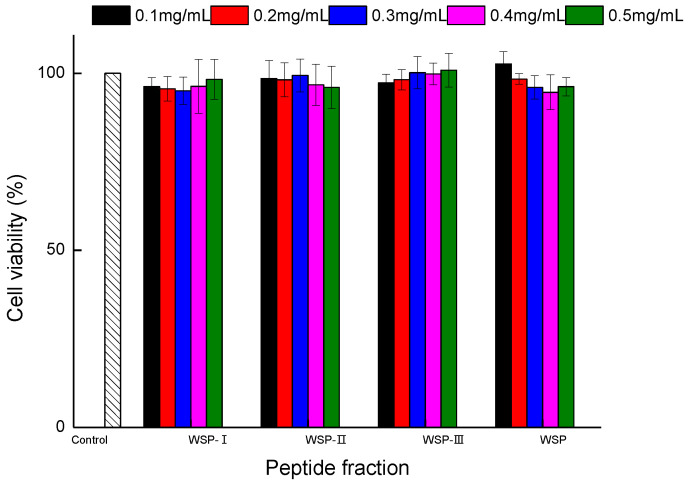
Effect of peptide fractions on viability of Caco-2 cells. Control: cells treated with medium only. Cells were pretreated with peptide fractions at 0.1–0.5 mg/mL for 24 h before exposure to MTT for 2 h. Data are expressed as mean ± standard deviation, *n* = 6. No significant difference was observed between each sample group and control group (*p* > 0.05).

The effects of WSP-Ⅰ, WSP-Ⅱ, WSP-Ⅲ, and WSP on cell viability were evaluated using the MTT assay. MTT assay is considered as the most commonly used and effective international method for detecting cell survival and growth [7]. The results are depicted in Figure 1, there was no significant difference between each peptide fraction group and control group (*p* > 0.05), and the cell survival rate of all groups was still above 90%. The results indicated that the five tested concentrations (0.1–0.5 mg/mL) of four peptide fractions would not evoke significant changes in the cell viability of Caco-2 cells and will not damage the integrity of the cells. This is consistent with the results of Huma et al. [4] and Rafiq et al. [7], who reported that the cheese peptides had no toxic effect on cell viability. Thus, the nontoxic concentrations (0.1–0.5 mg/mL) were used in the following study. 

### 3.2. Cytotoxicity Caused by H_2_O_2_

To determine the effect of H_2_O_2_ on cell viability, we treated the cells with H_2_O_2_ (0.2–1.1 mM) for 24 h. Cells treated with H_2_O_2_ showed significant symptoms of side effects, such as cell death and morphological changes, compared to controls. While H_2_O_2_ caused a decrease in cell viability in a concentration-dependent manner, the cell viability was significantly reduced to (55.38 ± 3.31)% when the concentration of H_2_O_2_ was 0.9 mM, and (28.16 ± 1.93)% when H_2_O_2_ was 1.1 mM (other data are not shown). H_2_O_2_ of 0.9 mM was chosen to induce the oxidative stress in Caco-2 cells throughout the present study for further analysis, as 0.9 mM H_2_O_2_ made the cell viability about half that of the control group.

### 3.3. Protective Effect of Cheese Peptide Fractions against H_2_O_2_-Induced Oxidative Stress in Caco-2 Cells

**Figure 2:**
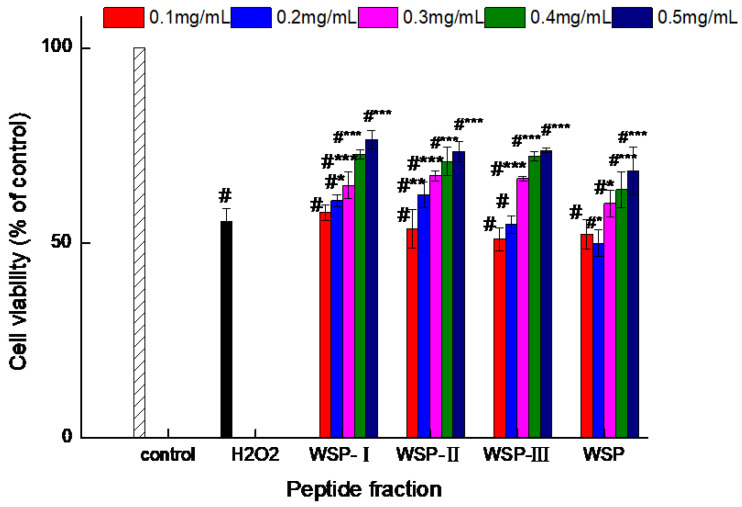
The protective effect of peptide fractions on viability (%) in H_2_O_2_-induced Caco-2 cells. Control: cells treated with medium only; H_2_O_2_: cells treated with medium and 0.9 mM H_2_O_2_. Values are expressed as the mean ± standard deviation, *n* = 6. # Extremely significant difference from the control group (*p* < 0.001); * Significant difference from the H_2_O_2_-induced group (*p* < 0.05); ** Significant difference from the H_2_O_2_-induced group (*p* < 0.01); *** Extremely significant difference from the H_2_O_2_-induced group (*p* < 0.001).

As the first method, the impact of WSP-Ⅰ, WSP-Ⅱ, WSP-Ⅲ, and WSP on the viability of cells injured by H_2_O_2_ was evaluated, and the results are shown in Figure 2. H_2_O_2_ reduced the viability of Caco-2 cells by 45% (*p* < 0.05) as compared to the control group, and all four peptide fractions exhibited the ability to enhance cell vitality of H_2_O_2_-induced cells. At low concentration (0.1–0.3 mg/mL), four peptide fractions had no statistically significant effect on viability, while higher concentrations of four peptide fractions (0.4 and 0.5 mg/mL) showed a significant (*p* < 0.05) protective effect against oxidative stress by increasing the cell viability to about 70% as compared to the group treated with H_2_O_2_ alone. These results supported the previous studies of Tonolo et al. [30] and Huma et al. [4], who found the protective effect of milk-derived peptides and cheese peptides on Caco-2 cell viability. In this study, the WSP-I showed the best protective effect, increasing cell viability to 76.47% at 0.5 mg/mL.

**Figure 3:**
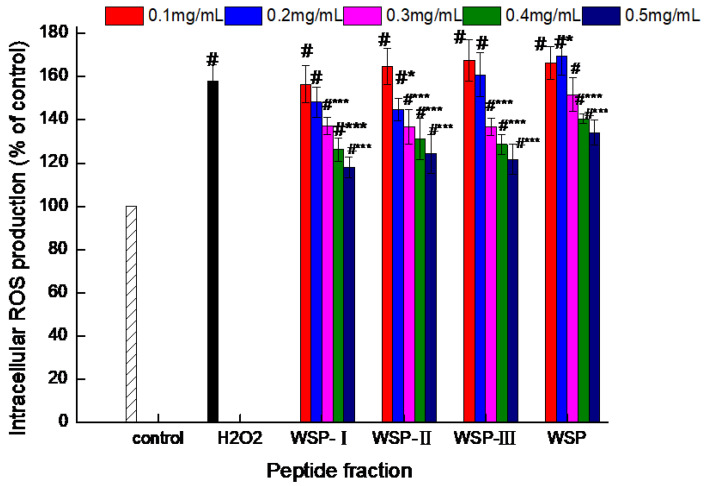
Effect of peptide fractions on intracellular ROS production (%) in H_2_O_2_-induced Caco-2 cells. Control: cells treated with medium only; H_2_O_2_: cells treated with medium and 0.9 mM H_2_O_2_. Values are expressed as the mean ± standard deviation, *n* = 6. # Extremely significant difference from the control group (*p* < 0.001); * Significant difference from the H_2_O_2_-induced group (*p* < 0.05); *** Extremely significant difference from the H_2_O_2_-induced group (*p* < 0.001).

Thereafter, the protective effect of WSP-Ⅰ, WSP-Ⅱ, WSP-Ⅲ, and WSP was evaluated by measuring the ROS quenching ability of four peptide fractions in oxidative-damaged cells. The DCFH-DA probe assay was used to determine the intracellular ROS-scavenging activity of the four peptide fractions. As shown in Figure 3, after exposure to H_2_O_2_, cells showed significantly increased ROS (*p* < 0.05) as compared to the control group and were oxidative damaged. Pretreatment with all four peptide fractions at 0.3–0.5 mg/mL for 24 h statistically decreased the intracellular ROS level (*p* < 0.05), with WSP-I displaying the best ROS-scavenging activity. The decrease in ROS levels indicates the ability of the four peptide fractions to protect oxidant-stressed Caco-2 cells. Huma et al. [4] also reported the antioxidant potential of cheddar cheese on human colon adenocarcinoma cells, and they noticed that cheese peptides protected oxidative-damaged cells to a certain extent by reducing the production of ROS in cells. Excessive accumulation of ROS is considered to be toxic to cells and may lead to the occurrence of various chronic diseases. Antioxidants can attenuate the occurrence of chronic diseases by reducing the accumulation of ROS. The soybean protein hydrolysate and the peptide fractions have been demonstrated to suppress intracellular ROS accumulation induced by H_2_O_2_ in Caco-2 cells [14]. Eggshell membrane hydrolysates have been reported to maintain vitality at the intestinal level and prevent oxidative damage [37]. Corn gluten meal-derived antioxidant peptides have been shown to have the inhibitory effect on H_2_O_2_-induced ROS generation [16]. 

### 3.4. Estimation of MDA Content, CAT and SOD Activity in Caco-2 Cells

Taking the above experimental results, the WSP-Ⅰ, WSP-Ⅱ, WSP-Ⅲ, and WSP at 0.4–0.5 mg/mL exhibited the higher cytoprotective effect against H_2_O_2_-induced damage, thus they were selected for further investigations. 

**Figure 4:**
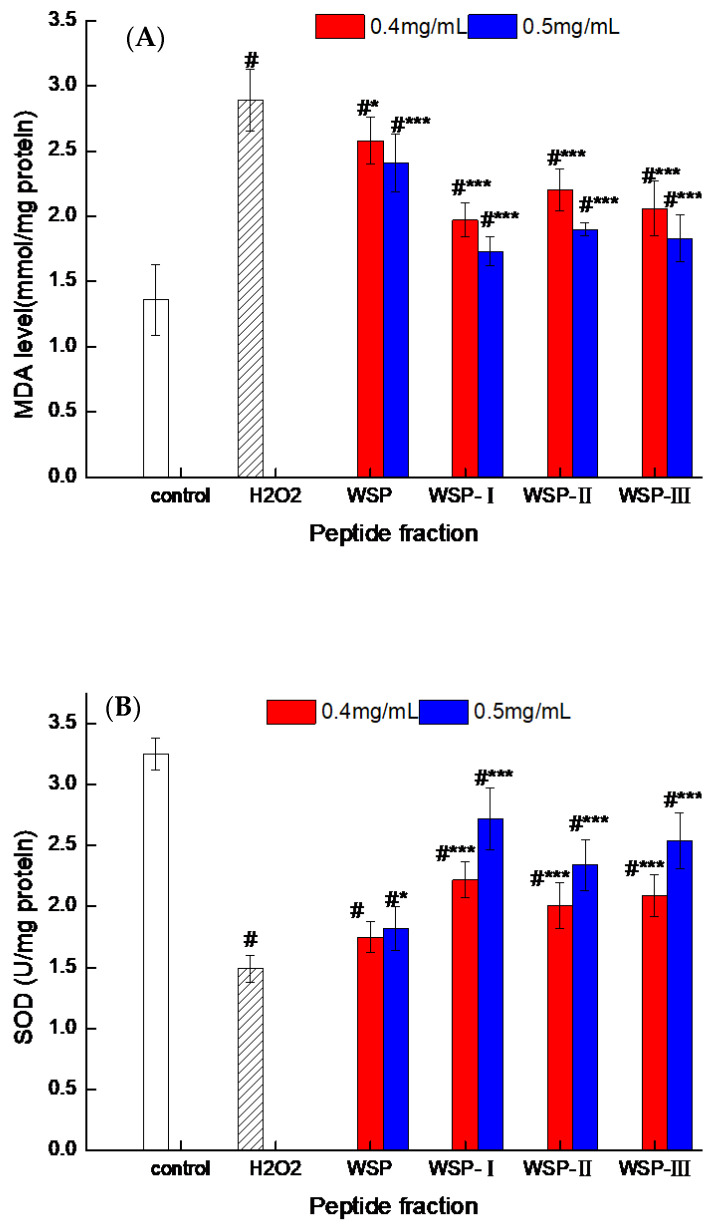

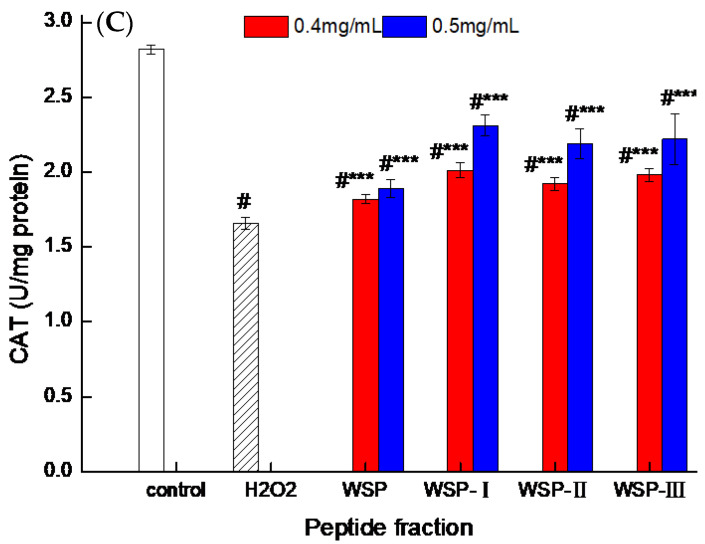
Effect of peptide fractions on MDA level (**A**), SOD activity (**B**), CAT activity (**C**) in Caco-2 cells. Control: cells treated with medium only; H_2_O_2_: cells treated with medium and 0.9 mM H_2_O_2_. Values are expressed as the mean ± standard deviation, *n* = 6. # Extremely significant difference from the control group (*p* < 0.001); * Significant difference from the H_2_O_2_-induced group (*p* < 0.05); *** Extremely significant difference from the H_2_O_2_-induced group (*p* < 0.001).

Firstly, the level of MDA was measured to evaluate the antioxidant capacity of WSP-Ⅰ, WSP-Ⅱ, WSP-Ⅲ, and WSP on oxidative-damaged cells. MDA is one of the final products of lipid peroxidation, which can be used as an effective biomarker for evaluating oxidative stress status [38]. As shown in Figure 4A, the level of MDA in Caco-2 cells treated with H_2_O_2_ alone was significantly increased in comparison to the control group, indicating the occurrence of lipid peroxidation in damaged cells. However, 24 h presupplementation of four peptide fractions at 0.4 mg/mL and 0.5 mg/mL significantly downregulated the overproduction of MDA in H_2_O_2_-induced cells (*p* < 0.05), indicating that four peptide fractions can attenuate cellular lipid peroxidation and ROS-mediated membrane damage. In addition, WSP-I displayed a higher inhibitory effect when compared to WSP-II, WSP-III, and WSP. 

In order to further clarify the cytoprotective effect of WSP-I, WSP-II, WSP-III, and WSP, their effects on cellular antioxidant enzyme activities, including those of SOD and CAT, were determined (Figure 4B,C). As seen, treatment with H_2_O_2_ significantly (*p* < 0.05) suppressed CAT and SOD activities compared to the control group, indicating that H_2_O_2_ could increase oxidative stress in Caco-2 cells. This was similar to the report by Garcia-Nebot et al. [39] and consistent with the view of Wijeratne et al. [40], who also found that oxidative damage resulted in the decrease of CAT and SOD activities. As seen in Figure 4B,C, after exposure to H_2_O_2_, the cellular CAT and SOD activities were markedly decreased by 54.15% and 41.13%, respectively, versus the control group, while peptide fractions significantly (*p* < 0.05) upregulated the cellular CAT and SOD activities by about 20% and 30%, respectively, as compared to the H_2_O_2_ group. Overall, the peptide fractions of the cheese contained the peptides that act as protective agents against H_2_O_2_-induced oxidative damage in Caco-2 cells. The protective effect of these peptides on oxidative-damaged cells may be due to their ability to reduce ROS in the cell, attenuate lipid peroxidation in the cell, and increase the activity of antioxidant enzymes. Likewise, an increase in CAT activity has been reported after treating Caco-2 cells for 24 h with alcalase-hydrolyzed soybean hydrolysate [14]. Guo et al. [32] also depicted a peptide from chickpea protein hydrolysates that protected Caco-2 cells from H_2_O_2_-induced damage via stimulating CAT activity. This same observation was also reported when Caco-2 cells were treated with buffalo casein-derived peptides, followed by oxidative stress with H_2_O_2_ [41]. Moreover, the activities of antioxidant enzymes increased with the increase of concentration of peptide fractions, which was consistent with the report by Wu et al. [42]. In our work, the highest SOD and CAT activities in Caco-2 cells were (2.72 ± 0.25) U mg/prot and (2.31 ± 0.07) U mg/prot, respectively, which were observed following treatment with 0.5 mg/mL of WSP-Ⅰ. These results indicated that the WSP-Ⅰ, WSP-Ⅱ, WSP-Ⅲ, and WSP have an attenuated effect on the reduction of CAT and SOD activities caused by H_2_O_2_. The increased activity of CAT and SOD indicates that the peptide fractions of cheese can respond to cellular oxidative damage. Similar results have been reported by Katayama et al. [43], who found that oligophosphopeptides of yolk phosphoprotein can upregulate antioxidant enzymes activities in Caco-2 cells against oxidative damage. These results are also similar to the previous results of buffalo casein peptides [41] and corn gluten meal peptides [2], that food-derived bioactive peptide fractions could increase the activity of antioxidant enzymes. Overall, our results suggest that the peptide fractions of cheese have a positive effect on the endogenous antioxidant enzyme activity in Caco-2 cells with oxidative damage. Based on the above results, WSP-Ⅰ was selected to further analyze the effect on oxidative damage cells.

### 3.5. Metabolomics Analysis

#### 3.5.1. Multivariate Data Analysis

**Figure 5:**
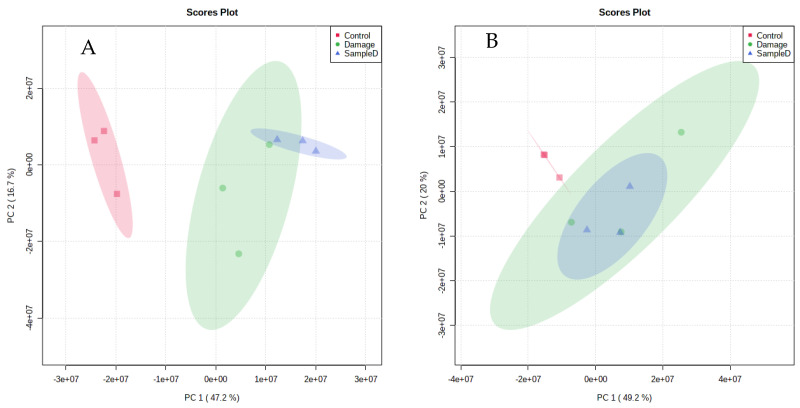

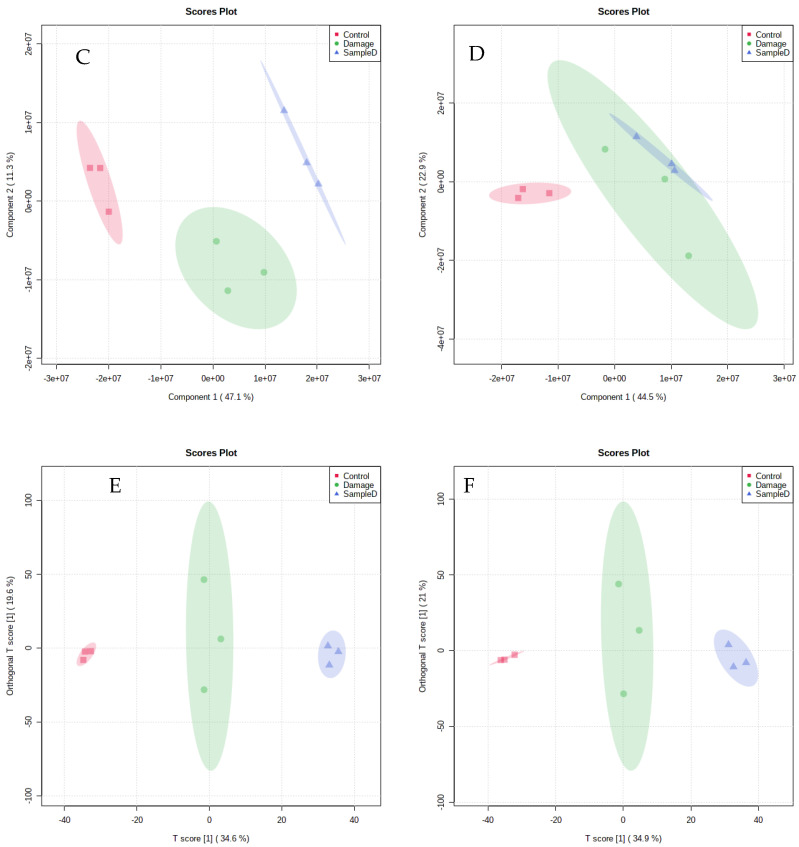
The score plot of PCA (**A**,**B**), PLS−DA (**C**,**D**), and OPLS−DA (**E**,**F**). (**A**,**C**,**E**) positive; (**B**,**D**,**F**) negative. Control: control group; Damage: damage group; SampleD: sample protection group.

The metabolomics data were first analyzed with the PCA multivariate model. As shown in Figure 5A,B, the control group could be obviously gathered in the positive and negative ion modes, while the damage group and the sample protection group had crossover and cannot be clearly distinguished, which showed that the effects of H_2_O_2_ and WSP-Ⅰ on the metabolic profile changed in Caco-2 cells. PCA can only reflect the original state of the metabolomics data and cannot eliminate the impact of environmental factors and systematic errors on the experimental results. Therefore, PLS-DA and OPLS-DA analysis models were used to further analyze the metabolomics data. The results of the PLS-DA analysis model were similar to that of PCA, and the damage group and sample protection group cannot be completely separated (Figure 5C,D). Figure 5E,F showed that the OPLS-DA model can effectively separate the control group, the damage group, and the sample protection group, and there was good aggregation within the group. R^2^X, R^2^Y, and Q^2^ were used to evaluate the OPLS-DA models. The closer the value of each indicator to 1, the more credible the model becomes. The R^2^X values were 0.346 and 0.349, the R^2^Y values were 0.908 and 0.874, and the Q^2^ values were 0.804 and 0.766 in the positive and negative ion modes, respectively. The above results indicated that H_2_O_2_ treatment caused changes in cell metabolites, and pretreatment with WSP-Ⅰ improved the changes in metabolites caused by H_2_O_2_.

#### 3.5.2. Identification of Significantly Different Metabolites 

VIP > 1 and *p* < 0.05 were rigorously used as the screening criteria for significantly different metabolites. Fifteen significantly different metabolites were identified, and their retention times, *m*/*z*, adducts, and cellular locations are summarized in Table 1. According to the human metabolome database, these metabolites are present in the extracellular, mitochondria, cytoplasm, nucleus, membrane, endoplasmic reticulum, or lysosome of the cell. 

**Figure 6:**
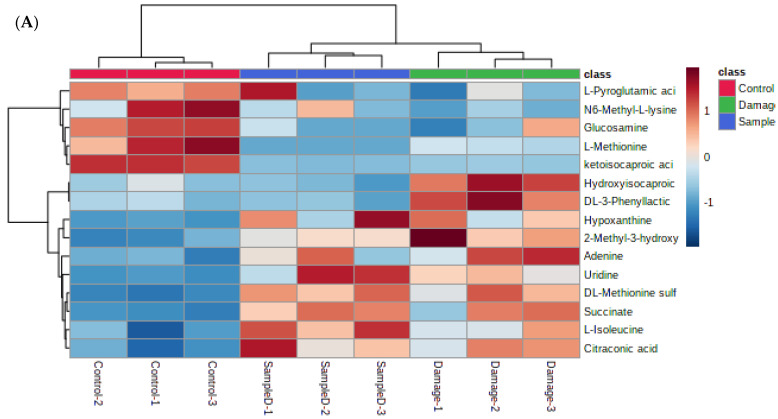

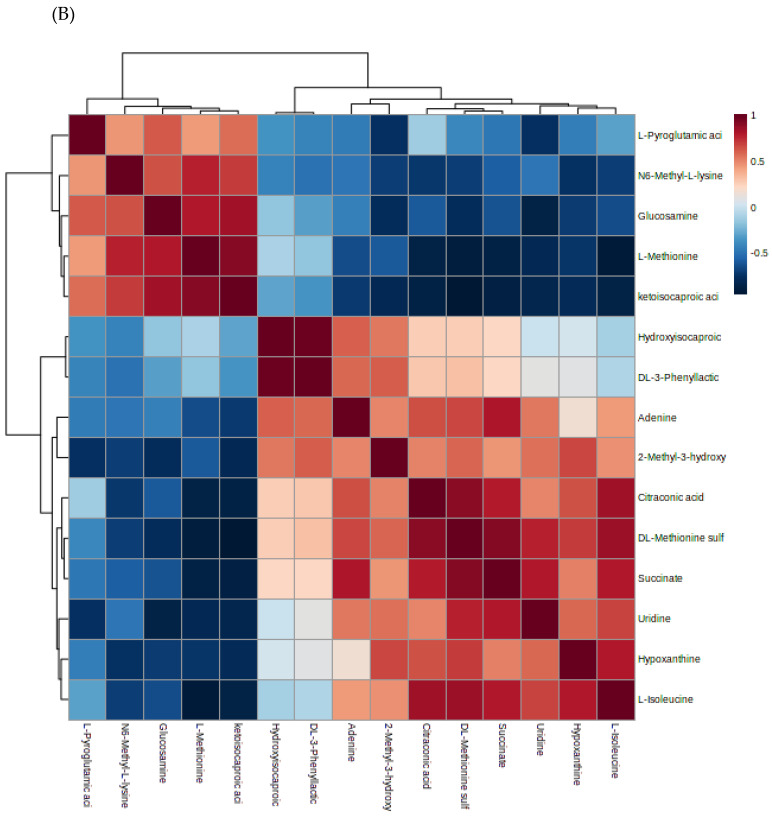
Hierarchical clustering heat maps of metabolites expression of different groups (**A**) and heat map of metabolite correlation (**B**). Control: control group; Damage: damage group; SampleD: sample protection group.

To further analyze the effects of H_2_O_2_ and WSP-Ⅰ on the 15 significantly different metabolites mentioned above, the heat maps were drawn in the analysis. The results of the expression levels of these 15 significantly different metabolites in three groups are shown in Figure 6A. Red indicates that the metabolite content was upregulated, and blue indicates that the expression of metabolite was downregulated. The darker the color, the greater the degree of upregulation/downregulation. Pretreatment with H_2_O_2_ downregulated five metabolites including L-methionine, ketoisocaproic acid, L-pyroglutamic acid, N6-methyl-L-lysine, and glucosamine. Ten metabolites were upregulated, including uridine, DL-methionine sulfoxide, hypoxanthine, adenine, L-isoleucine, hydroxyisocaproic acid, DL-3-phenyllactic acid, succinate, citraconic acid, and 2-methyl-3-hydroxybutyric acid. Pretreatment with WSP-Ⅰ improved the change of metabolite expression content caused by H_2_O_2_. At the same time, levels of adenine, hydroxyisocaproic acid, DL-3-Phenyllactic acid, and 2-methyl-3-hydroxybutyric acid decreased significantly, while levels of L-pyroglutamic acid and N6-methyl-L-lysine increased significantly in the sample group compared to the damage group.

#### 3.5.3. Pathway Analysis

To identify the metabolic pathway by which WSP-Ⅰ interfere with cell oxidative damage, the correlation between 15 significantly different metabolites was analyzed. The results are shown in Figure 6B. It can be seen that the abovementioned 15 metabolites had positive or negative correlation with each other, indicating that the protective effect of WSP-Ⅰ on oxidative-damaged cells may be related to multiple metabolic pathways, and these significantly different metabolites and metabolic pathways interact with each other.

**Figure 7:**
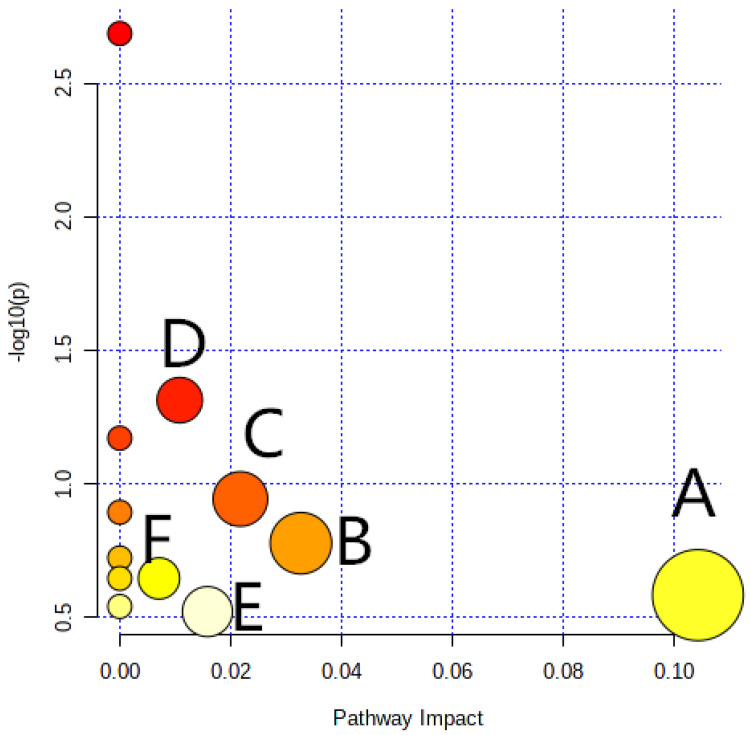
Overview of pathway analysis from pathway topology analysis. A: Cysteine and methionine metabolism; B: Citrate cycle (TCA cycle); C: Purine metabolism; D: Valine, leucine, and isoleucine degradation; E: Pyrimidine metabolism; F: Glutathione metabolism.

**Figure 8:**
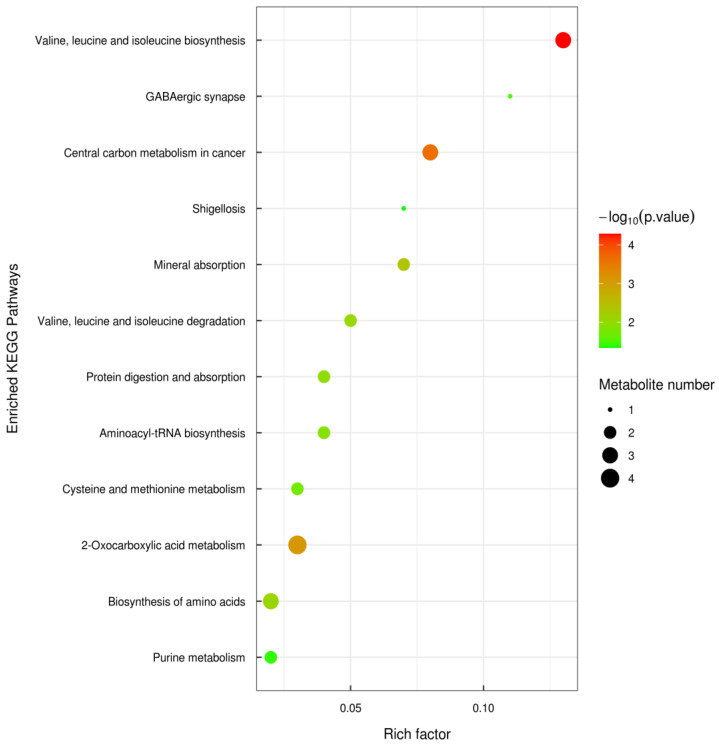
KEGG enrichment pathway map (bubble map).

A pathway analysis was performed on 15 of the abovementioned significantly different metabolites to determine biologically significantly metabolic patterns and associated pathways. The results for the pathway topology are shown in Figure 7, and the results for the enrichment of the KEGG pathway are shown in Figure 8. According to the pathway impact value and *p* value, it can be known that the pathways involved in the protective effect of WSP-Ⅰ on oxidative-damaged cells were mainly cysteine and methionine metabolism, citrate cycle (TCA cycle), purine metabolism, valine, leucine, and isoleucine degradation, pyrimidine metabolism, glutathione metabolism, valine, leucine and isoleucine biosynthesis, central carbon metabolism in cancer, and 2-Oxocarboxylic acid metabolism.

In this study, L-pyroglutamic acid may be associated with glutathione metabolism. The level of L-pyroglutamic acid was decreased in the damage group and elevated after the treatment with WSP-Ⅰ. Glutathione plays an important role in the nutrient metabolism, antioxidant defense, and immune response of organisms [35,44]. A decrease in the level of L-pyroglutamate acid in the damage group cells indicates that the cells may have been damaged by oxidation. In the sample protection group, the content of L-pyroglutamic acid increased, indicating the preventive effect of the WSP-Ⅰ against oxidative damage [4]. Succinate was an intermediate product of tricarboxylic acid recycling (TCA). The WSP-Ⅰ regulated the succinate in the cells and improved the disturbed energy metabolism of the cells. DL-methionine sulfoxide is involved in cysteine and methionine metabolism, and ketoisocaproic acid and L-isoleucine may be associated with valine, leucine, and isoleucine degradation. Ketoisocaproic acid, L-isoleucine, citraconic acid, and DL-methionine are involved in the metabolism of amino acids in cells. Amino acids play an important role in the material metabolism and energy metabolism of organisms, and were the synthetic raw materials for various biologically active substances such as proteins [45,46,47]. Isoleucine was a ketogenic and sugar-generating amino acid, which could produce both succinyl-CoA and acetyl-CoA to participate in the tricarboxylic acid cycle to provide energy for cells [48,49]. An abnormal change in the content of isoleucine in cells damaged by H_2_O_2_ indicates that the cell’s carbohydrate metabolism and ketone metabolism are disturbed, as well as its energy metabolism. In the sample protection group, the abnormal expression of these metabolites was improved, proving the protective effect of WSP-Ⅰ on oxidative-damaged cells.

## 4. Conclusions

The current results of this study demonstrate that the peptide fractions of cheddar cheese made with *Lactobacillus helveticus* 1.0612 alleviates the oxidative damage induced by H_2_O_2_ and modulates abnormal ROS, MDA, and SOD levels to normal levels. It also weakens the metabolic disorders caused by H_2_O_2_ by regulating energy metabolism and amino acid metabolism. Taken together, the results of the current study data suggest that the peptide fractions of cheese may be valuable in protecting against oxidative stress. Thus, the biological function of cheese merits further investigation.

## Figures and Tables

**Table 1:** Significantly different metabolites.

Mode	Adduct	Name	*m*/*z*	rt (s)	Cellular Location
Positive	(M+H)+	Uridine	245.07621	280.438	Extracellular; Mitochondria; Nucleus; Lysosome
	(M+H)+	DL-Methionine sulfoxide	166.05303	671.815	Cytoplasm
	(M+H)+	L-Methionine	150.05817	478.758	Extracellular
	(M+H)+	L-Pyroglutamic acid	130.05014	793.963	Cytoplasm
	(M+H)+	Hypoxanthine	137.04595	280.6695	Cytoplasm; Extracellular; Lysosome; Peroxisome
	(M+H)+	Adenine	136.06202	271.4855	Cytoplasm; Extracellular; Nucleus; Lysosome
	(M+H)+	L-Isoleucine	132.1023	461.152	Cytoplasm; Extracellular; Mitochondria
	(M+H)+	N6-Methyl-L-lysine	161.12801	1007.5	Cellular Locations Not Available
Negative	(M-H) −	ketoisocaproic acid	129.05732	72.204	Cytoplasm; Extracellular; Membrane; Mitochondria
	(M-H) −	Hydroxyisocaproic acid	131.0723	75.947	Extracellular; Membrane
	(M-H) −	DL-3-Phenyllactic acid	165.0564	73.4145	Membrane
	(M-H) −	Citraconic acid	129.02036	109.1885	Extracellular; Membrane
	(M-H) −	2-Methyl-3-hydroxybutyric acid	117.05726	210.044	Cytoplasm
	(M-H) −	Succinate	117.02113	690.081	Extracellular; Mitochondria; Endoplasmic reticulum; Peroxisome
	(M+CH3COO) −	Glucosamine	238.09241	651.2315	Cytoplasm; Extracellular; Membrane

## Data Availability

Data sharing not applicable. No new data were created or analyzed in this study. Data sharing is not applicable to this article.
